# The damage and remineralization strategies of dental hard tissues following radiotherapy

**DOI:** 10.1186/s12903-024-04561-7

**Published:** 2024-07-16

**Authors:** Lin Yao, Yanyao Li, Di Fu, Ye Wang, Chengge Hua, Ling Zou, Li Jiang

**Affiliations:** 1 State Key Laboratory of Oral Diseases, West China Hospital of Stomatology, National Clinical Research Center for Oral Diseases, Sichuan University, Chengdu, 610041 China; 2 State Key Laboratory of Oral Diseases, Department of General Dentistry, West China Hospital of Stomatology, National Clinical Research Center for Oral Diseases, Sichuan University, Chengdu, 610041 China; 3 State Key Laboratory of Oral Diseases, Department of Endodontics, West China Hospital of Stomatology, National Clinical Research Center for Oral Diseases, Sichuan University, Chengdu, 610041 China

**Keywords:** Radiotherapy, Radiation-related caries, Dental hard tissue remineralization, Titanium tetrafluoride, Casein phosphopeptide-amorphous calcium phosphate

## Abstract

**Objectives:**

This study pursued two main purposes. The first aim was to expound on the microscopic factors of radiation-related caries (RRC). Further, it aimed to compare the remineralization effect of different remineralizing agents on demineralized teeth after radiotherapy.

**Methods:**

The enamel and dentin samples of bovine teeth were irradiated with different doses of radiation. After analysis of scanning electron microscope (SEM), X-Ray diffraction (XRD), and energy dispersive spectrometer (EDS), the samples irradiated with 50 Gy radiation were selected and divided into the demineralization group, the double distilled water (DDW) group, the Sodium fluoride (NaF) group, the Casein phosphopeptide-amorphous calcium phosphate (CPP-ACP) group, the NaF + CPP-ACP group, and the Titanium tetrafluoride (TiF_4_) group. After demineralization, remineralizing agents treatment, and remineralization, the samples were evaluated using SEM, atomic force microscope (AFM), EDS, and transverse microradiography (TMR).

**Results:**

A radiation dose of 30 Gy was sufficient to cause damage to the dentinal tubules, but 70 Gy radiation had little effect on the microstructure of enamel. Additionally, the NaF + CPP-ACP group and the TiF_4_ group significantly promoted deposit formation, decreased surface roughness, and reduced mineral loss and lesion depth of demineralized enamel and dentin samples after radiation.

**Conclusions:**

Radiation causes more significant damage to dentin compared to enamel. NaF + CPP-ACP and TiF_4_ had a promising ability to promote remineralization of irradiated dental hard tissues.

**Advances in knowledge:**

This in vitro study contributes to determining a safer radiation dose range for teeth and identifying the most effective remineralization approach for RRC.

## Introduction

Head and neck cancer (HNC) standing as the seventh most common malignancy in the world [1, 2], mainly occurs at sites including the oral cavity, nasopharynx, oropharynx, hypopharynx, larynx, and trachea [3]. Radiotherapy is of vital importance in HNC management [4]. Despite enhancing patient survival rates, it often results in severe tissue damage [5], and the early or late adverse events stemming from radiotherapy of HNC are difficult to control. Radiation may have implications for various components within the oral cavity, including the oral mucosa, salivary glands, bones, masticatory muscles, and dentition of afflicted individuals. Early-stage radiotherapy often manifests with symptoms such as taste loss and diminished salivary gland function, directly or indirectly contributing to radiation-related caries (RRC) [6].

RRC is an invasive condition that occurs in HNC patients undergoing radiotherapy. One of the typical features of RRC is that lesions start on the cervical areas of labial surfaces, progress around the tooth cervical areas [6], and form a“cervical ring” [7]. Radiation can cause changes in the chemical composition, especially the organic component, of dental hard tissues, thereby altering their biomechanical properties. Moreover, due to the higher content of organic components in the enamel-dentinal junction (EDJ) and dentin, the impact of radiation on EDJ and dentin is relatively greater compared to enamel [8, 9]. Radiotherapy may also have an impact on the polymerization and bonding of resins, making subsequent dental restorations challenging [8]. If not diagnosed and treated in time, RRC will lead to tooth fractures [10], or even tooth loss [11]. At present, there is no widely acknowledged clinical treatment for effectively repairing RRC with a high success rate. Hence, the prevention and management of RRC are necessary to enhance the quality of life for HNC patients following radiotherapy [12, 13].

To prevent RRC, we should first understand the etiology, and the damage of enamel and dentin caused by radiation is an internal factor. Kudkuli et al. argued that radiotherapy caused substantial alterations in both the inorganic and organic functional groups on the enamel surface [14]. Radiation doses exceeding 40 Gy could reduce the microhardness of enamel and dentin, affecting their bonding capacity [15]. Conversely, Gonçalves et al. suggested that radiation did not directly affect the inorganic structure of human teeth or alter the overall microhardness of enamel. The observed changes in enamel’s physical properties after radiotherapy are attributed to alterations in the organic matrix within the enamel [16]. Lu et al. found that the protein-to-mineral ratio in enamel increased following cumulative radiation, while the ratio in dentin decreased [17]. Although a lot of studies showed the effect of gamma radiation on teeth, their views were quite different. Thus, the effect of radiation on dental hard tissues needs further study.

Demineralization and remineralization of dental hard tissues in the oral cavity are always alternating. However, under pathological conditions, demineralization is stronger than remineralization [18], natural remineralization alone is not enough [19]. To manage RRC, promoting tooth remineralization is an effective non-invasive treatment method. Fluorid-based strategies remain the standard for caries prevention and management [20]. Sodium fluoride (NaF) is a widely used anti-caries agent in clinical practice nowadays. The application of fluoride varnish for three months has been shown to effectively reduce the incidence of RRC [21]. It is reported that the remineralization effect of fluoride is dose-dependent, toothpaste containing 5000 ppm fluoride is more effective for remineralization of root caries lesions than toothpaste containing 1000 to 1500 ppm fluoride [22]. Therefore, a supplement or alternative medicine is needed to control the dose of fluoride and enhance fluoride effects, especially in high-caries-risk individuals and groups of people [19].

Casein phosphopeptide-amorphous calcium phosphate (CPP-ACP) is a novel nano-scale biological anti-caries agent derived from milk protein, which is probably the most studied non-fluoride remineralizing agent [19]. Some reviews suggested that the quantity and quality of clinical trial evidence were insufficient to demonstrate the long-term effectiveness of CPP-ACP in preventing dental caries in vivo [23, 24]. Recently, shen et al. demonstrated that the inclusion of 5% CPP-ACP in yogurt can enhance enamel caries remineralization [25], and Sim et al. [60] corroborated that toothpaste enriched with NaF and CPP-ACP can enhance the bioavailability of calcium and phosphorus ions, balance the demineralization/remineralization process, and play a positive role in preventing the formation of cervical caries. Moreover, NaF and CPP-ACP have demonstrated their efficacy in halting the progression of root caries [26]. The potential of titanium tetrafluoride (TiF_4_) to prevent tooth demineralization has been investigated since 1997 [27]. After the treatment of demineralized bovine teeth with TiF_4_, the uptake of element F in enamel was higher than that of NaF [28]. A clinical trial employing 4% TiF_4_ for the prevention of childhood caries indicated that TiF_4_ exhibits comparable effectiveness and acceptability in controlling dental caries when compared to NaF [29]. Furthermore, some research indicated that NaF and TiF_4_ can mitigate dental hard tissue demineralization following radiation exposure and decelerate caries progression [30]. Nevertheless, limited research has reported the application of these remineralizing agents in the context of promoting dental hard tissue remineralization post-radiotherapy.

To verify the results of previous experiments on the effect of radiation on teeth, and to select a dose that was consistent with clinical practice and significantly increased the risk of RRC for subsequent experiments, we investigated the impact of different radiotherapy doses on the chemical composition and microstructure of tooth enamel and dentin. Additionally, this study compared the effects of diverse remineralization protocols on demineralized bovine teeth enamel and dentin following radiation exposure by scanning electron microscope (SEM), atomic force microscope (AFM), energy dispersive spectrometer (EDS), and transverse microradiography (TMR), aiming to identify the most effective remineralization approach. The null hypotheses were that different doses of radiation had no difference in their effects on the chemical composition and microstructure of dental hard tissues, and the remineralization effect of TiF_4_ and NaF + CPP-ACP on dental hard tissues demineralized after radiotherapy was not different from that of NaF or CPP-ACP used individually.

## Materials and methods

**Fig. 1:**
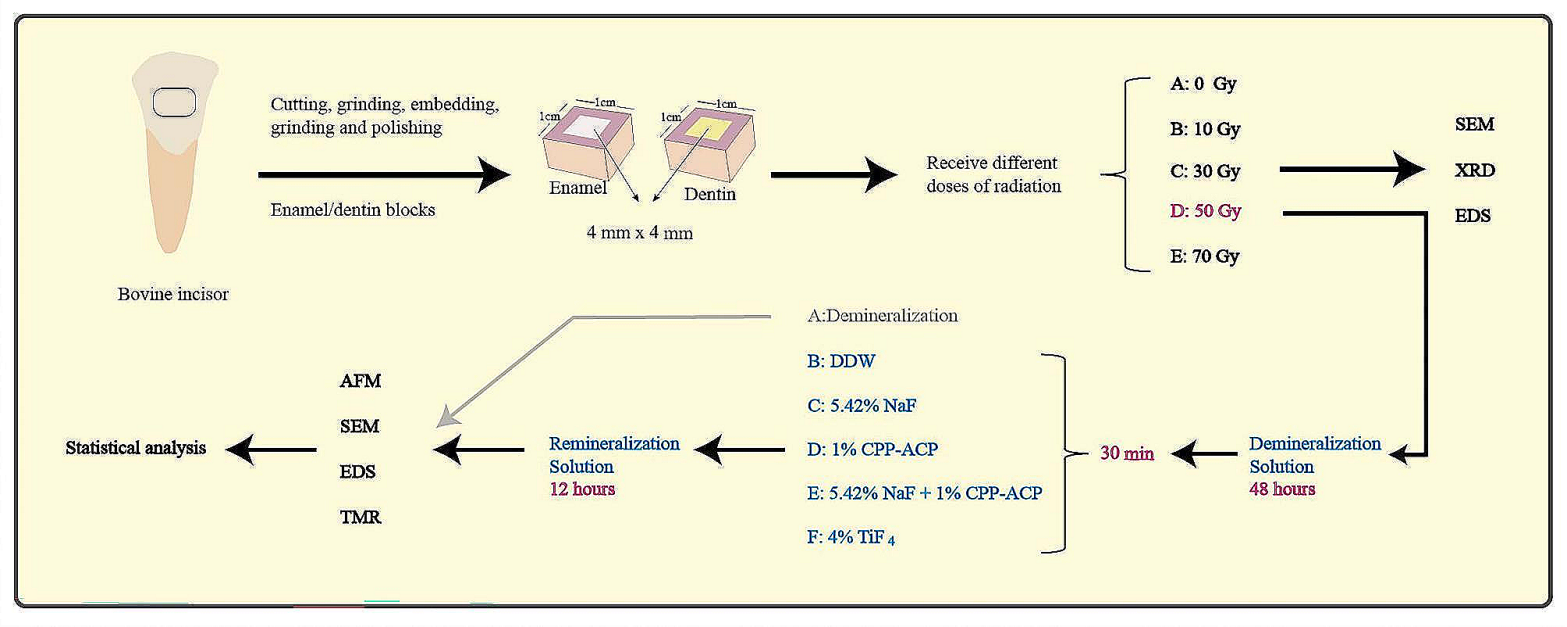
The flowchart of the experimental procedure

The flow of experimental methods is shown in Fig. 1.

### Materials

Thymol and polymethyl methacrylate were purchased from Macklin (Shanghai, China). NaF, CPP, ACP, and TiF_4_ were provided by Macklin (Shanghai, China). In our experiments, the remineralizing agents we used were prepared by dissolving the reagent powder in double distilled water (DDW) to create solutions of the required concentrations, Specifically, the CPP-ACP solution was prepared by mixing equal volumes of 1% CPP solution and 1% ACP solution.

### Sample preparation

The required sample size was determined using G*Power 3.1 software. Based on the findings from Turjanski et al. [31], the sample size per group in the first experiment was 4 (20 samples total) with a probability higher than 80%, a statistically significant difference of at least 15%, and a 0.05 significance level. According to the research of Parisay et al [32], The resulting sample size in our second experiment was 10 per group with an effect size of 0.5, a power of 0.8, and a significance level of 0.05. We prepared 13 samples per group (78 samples total) to compensate for laboratory errors.

Incisors of 5-year-old cows purchased from the local slaughterhouse were used in the study. The bovine permanent incisors were soaked in 0.1% thymol solution (Macklin, Shanghai, China) [28], and then the soft tissues and debris on the surface were removed by surgical instruments and an ultrasonic cleaner (FS20; Fisher Scientific Co., Pittsburgh, USA). Intact bovine teeth were divided into enamel blocks and dentin blocks with uniform size by a slow precision cutting machine (Minitom; Struers, Copenhagen, Denmark) and emery cutting saw blade (EXAKT300; EXAKT, Norderstedt, Germany) under running deionized water. The prepared enamel and dentin blocks were embedded in polymethyl methacrylate (Macklin, Shanghai, China) in a square mold of 1 cm × 1 cm, then the sample blocks were ground and polished with 300, 800, 1200, and 1500-grit waterproof silicon carbide paper (Yu Ying, Foshan, China) in water-cooled carborundum discs (Struers Minitom; Struers, Copenhagen, Denmark), and the surfaces of enamel and dentin were exposed to at least 4 × 4 mm^2^. Finally, 98 enamel samples and 98 dentin samples were obtained, which were soaked in PBS solution and stored in a refrigerator at 4 ℃.

### Exploring the impact of different doses of radiation on the microstructure and chemical composition of enamel and dentin

#### Sample irradiation

Twenty enamel samples and twenty dentin samples were randomly divided into five groups: 0 Gy (Control group), 10 Gy, 30 Gy, 50 Gy, and 70 Gy group (*N* = 4). The medical linear accelerator (Synergy; Elekta, Stockholm, Sweden) was used to irradiate the samples, the irradiation distance was 100 cm (from the window surface), the irradiation field was 20 × 20 cm^2^, and the irradiation power was 2.5 Gy/min. We used fractionated radiation, and all samples were within the pre-set radiation field range. To prevent machine overload, samples were irradiated for 2 minutes each time and then cooled for 10 min, followed by the next round of irradiation. Except for the control group, samples of the other four groups received corresponding radiation doses of 10 Gy, 30 Gy, 50 Gy, and 70 Gy, respectively by controlling the irradiation time.

#### Morphological characterization

Enamel and dentin samples were cut and polished into 4 × 4 × 2 mm^3^ sample blocks. After ultrasonic cleaning, they were dehydrated by 25%, 50%, 75%, and 95% ethanol solution in turn. Then, these samples were placed in a vacuum gold plating machine and sprayed with gold [33]. Finally, SEM (Apreo 2; Thermo Fisher Scientific, Brno, Czech) was performed to observe the microstructure of enamel and dentin at a magnification of 5000 × and 40,000 ×, a working distance of 10 ± 1 mm, and an accelerating voltage of 20 kV.

#### Element analysis

After treatment with the experimental steps described above for SEM, the appropriate areas in samples were selected in the scanning area of SEM, and the line scanning was carried out by EDS (X-MaxN; Oxford instrument, Oxford, UK) [34]. The measured data were compared and analyzed after a smooth line diagram, and the content and distribution of Ca, P, and C elements in enamel and dentin were detected.

#### Structural analysis

Through cutting and grinding, the samples with large and uniform windows were selected in each group. XRD (X’pert PRO; Malvern PANalytical, Almelo, Netherlands) measurements were performed, using CuKα radiation, to scan and record these samples in the range of 2θ (i.e. 10–60° in 0.01° step). The final XRD results were analyzed by HighScorePlusversion 5.1 (Malvern PANalytical, Almelo, Netherlands) to detect the crystal structures in enamel and dentin [35].

### Exploring the remineralization effect of different remineralizing agents on demineralized dental hard tissues after radiation

After a comprehensive analysis of the results of *experiment 2.2*, we chose a radiation dose of 50 Gy for the next experiment.

#### Sample irradiation

Seventy-eight enamel samples and seventy-eight dentin samples were irradiated with 50 Gy radiation according to the method above.

#### Processing of sample blocks

The sample blocks were placed in a demineralizing solution (2.2 mM KH_2_PO_4_, 2.2 mM Ca(NO_3_)_2_, 5.0 mM NaN_3_, 0.5 ppm NaF, 50 mM acetic acid, pH 4.5) at 37 ℃ for 48 h [37]. The demineralized sample blocks were washed with DDW for 2 min and then dried. Enamel and dentin samples were randomly divided into 6 groups (*N* = 13) and treated as follows: (1) demineralization group: no treatment, (2) DDW group: samples treated with DDW for 30 min, (3) NaF (Macklin, Shanghai, China) group: samples treated with 5.42% NaF solution for 30 min, (4) CPP-ACP (Macklin, Shanghai, China) group: samples treated with 1% CPP-ACP solution for 30 min, (5) NaF + CPP-ACP group: samples treated with a mixed solution of 5.42% NaF and 1% CPP-ACP for 30 min, (6) TiF_4_ (Macklin, Shanghai, China) group: samples treated with 4% TiF_4_ solution for 30 min. The remineralizing agents were brushed onto the sample surface three times with micro brushes (Jaan, Guangzhou, China), with each application repeating after a 10-minute interval. This ensured that the sample surface remained moist for a total of 30 min. The treatment groups (2)-(6) were washed with DDW for 2 min, then placed in remineralizing solution (20 mM HEPES, 0.9 mM KH_2_PO_4_, 1.5 mM CaCl_2_, 130 mM KCl, pH 7.0) at 37 ℃ for 12 h [36]. After finishing, the surface was washed with DDW for 2 min and dried for subsequent experiments.

#### SEM observation

Samples from each group were cut and polished into 4 × 4 × 2 mm^3^ sample blocks. The following experimental methods were consistent with those in 2.3.2. SEM was operated for scanning and image acquisition.

#### AFM observation

After demineralization and remineralization, samples from each group were scanned by AFM (SPM9700; Shimadzu, Kyoto, Japan), with a scan area of 5 × 5 µm^2^ and a scanning frequency of 1 Hz. Shimadzu SPM-9700 software (Shimadzu, Kyoto, Japan) was used to analyze the images, and the surface roughness (Ra) of each sample was calculated. Finally, statistical analysis was carried out [34].

#### EDS examination

The following experimental methods were consistent with those in 2.2.3. The changes of Ca, P, C, fluorine (F), and titanium (Ti) elements in the range of 80 μm from the sample surface were analyzed.

#### TMR examination

Samples were cut into 1 mm-thick slices perpendicular to the window opening surfaces with a slow-cutting machine. All the thin slices were polished to a thickness of 100–120 μm via 1500 grit carbide-polishing papers (Yu Ying, Foshan, China) [37], and the vernier caliper (Mitutoyo, Tokyo, Japan) was used to confirm that the thickness reached the standard. Each slice was then fixed on a TMR imaging slide (Konica Minolta, Tokyo, Japan) and micro-radiographed alongside an aluminum calibration stepwedge with 11 steps for 30 min with a monochrome CuK X-ray source (voltage: 20 kV, current: 20 mA, working distance: 40 cm). Photo plates were developed and fixed according to standard procedures. Images were collected using a transmitted light microscope with a 20 × objective (Zeiss, Oberkochen, Germany), which was equipped with a CCD camera (Canon, Tokyo, Japan) and connected to a computer (TOSHIBA, Tokyo, Japan). Three non-overlapping areas were selected for each sample, and the mineral loss and lesion depth of the samples were calculated using TMR Software 2006 (Inspektor Research Systems, Amsterdam, Netherlands) [38].

### Statistical analysis

Data were analyzed using SPSS 26.0 (IBM; Armonk, New York, USA). Shapiro-Wilk and Levene tests were used to verify the normal distribution and homogeneity of variance of data, respectively. The roughness data (Ra) followed a normal distribution and met the homogeneity of variance, so ANOVA variance analysis was used to test the results, and LSD analysis was used for pairwise comparisons. However, since the data on the mineral loss and the lesion depth could not simultaneously satisfy the normal distribution and homogeneity of variance, the Kruskal-Wallis H test was used to statistically analyze these data. Finally, the median ± quartile interval was used to represent the obtained data, and the test level α = 0.05.

## Results

### The changes in microstructure and chemical composition of dental hard tissues after different doses of irradiation

#### The microstructure and chemical composition of enamel after radiation

**Fig. 2:**
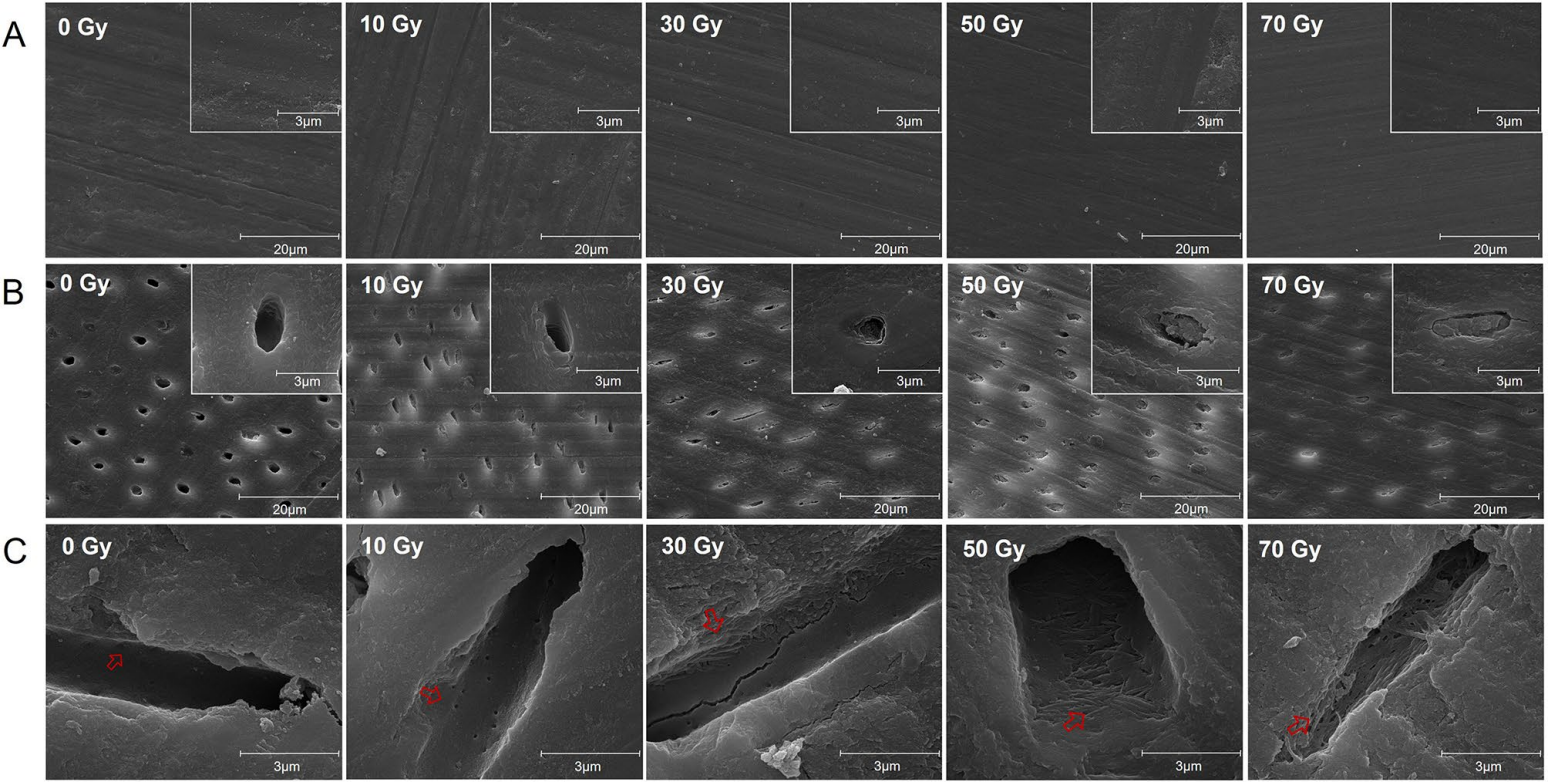
(**A**) Representative microstructure of enamel and (**B**) dentin after different doses of radiation (SEM images, 5000 × and 40,000 ×). (**C**) Representative longitudinal section of dentin after different doses of irradiation (SEM images, 40,000 ×). The red arrows indicate typical collagen fiber morphology

**Fig. 3:**
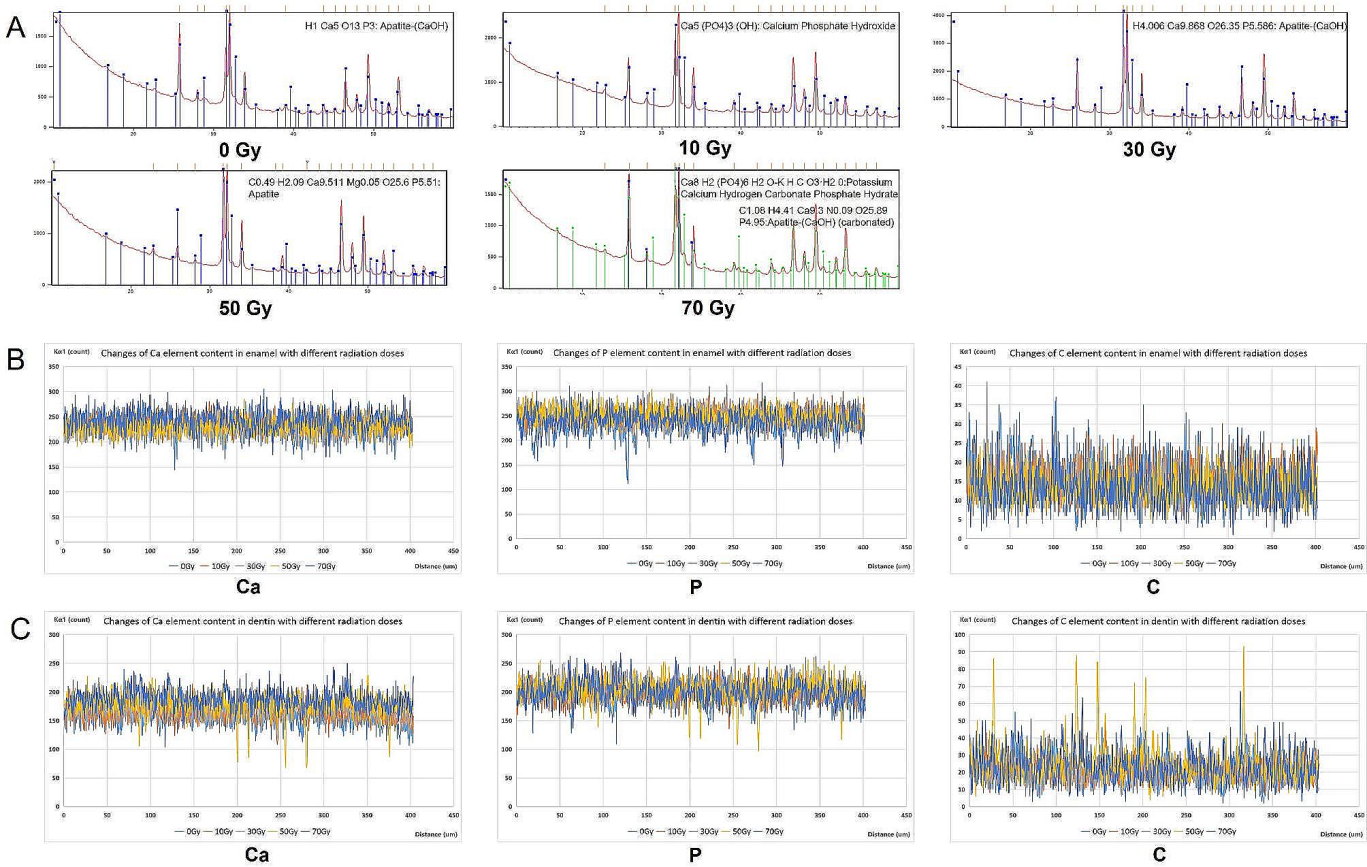
(**A**) The XRD phase analysis of enamel after different doses of radiation. (**B**) The content of Ca, P, and C elements in enamel and (**C**) dentin at different depths under different radiation doses

In this experiment, different doses of radiation did not affect the microstructure of the enamel surface (Fig. 2A). XRD phase analysis of enamel showed that hydroxyapatite crystals, composed of Ca, P, O, and H elements, were the main crystals in enamel at radiation doses of 0 Gy, 10 Gy, and 30 Gy. When radiation doses increased to 50 Gy, apatite crystals were composed of Ca, P, O, and H elements plus a small amount of Na, Mg, and C elements. When the radiation dose rose to 70 Gy, the content of C element in apatite crystals increased further, and non-apatite crystals, such as potassium calcium and bicarbonate phosphate, appeared at the same time (Fig. 3A).

#### The microstructure and chemical composition of dentin after radiation

According to the images of SEM, changes were found in the microstructure of dentin. Open dentinal tubules and dense and uniform surfaces in tubules could be seen in groups 0 Gy and 10 Gy, while dentinal tubules gradually collapsed and blocked with the increase of radiation dose (Fig. 2B). From the longitudinal section of dentin tubules, it could be seen that when the radiation dose reached 30 Gy, the inner wall of dentin tubules was no longer smooth and homogeneous, and the dentin collagen fibers became sparse. With the radiation dose increasing to 70 Gy, dentin collagen fibers became disordered from orderly interweaving and obvious fracture could be found (Fig. 2C).

#### Elemental content of dental hard tissues after radiation

According to the results of EDS, the contents of Ca, P, and C elements in different depths fluctuated slightly, but there was no obvious difference in the contents of the three elements in enamel (Fig. 3B) and dentin (Fig. 3C) treated by different doses of radiation.

### Comparison of the remineralization effects of different remineralizing agents

#### Changes in surface roughness of dental hard tissues after remineralization

**Fig. 4:**
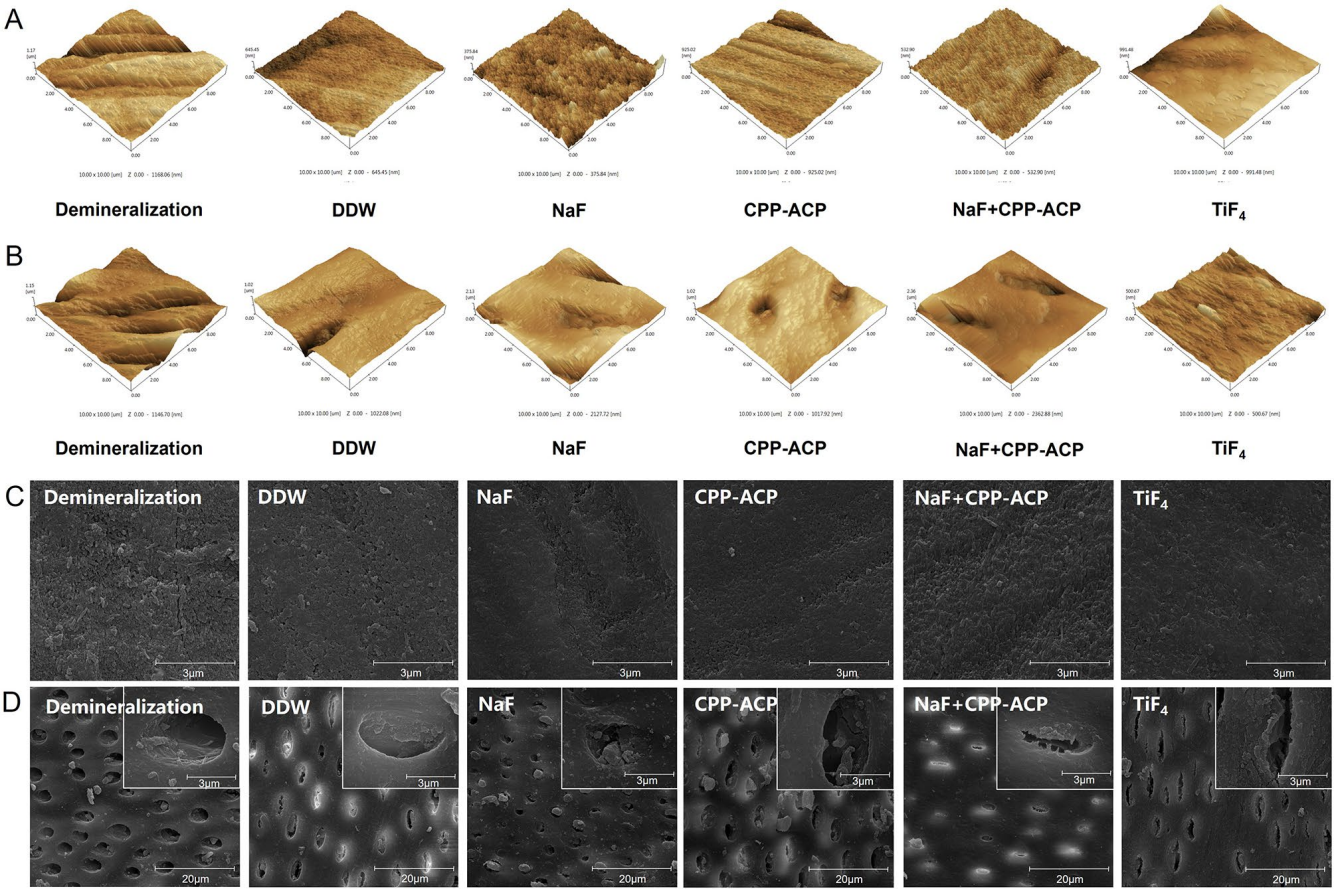
(**A**) Representative AFM images of enamel and (**B**) dentin surfaces in each group. (**C**) Representative SEM image of enamel in each group (5000 ×). (**D**) Representative SEM image of dentin in each group (5000 × and 40,000 ×)

We used AFM to examine the surface roughness of the samples. In the DDW group and the CPP-ACP group, the surface of the enamel showed obvious erosion marks and low smoothness. Dense cluster deposits were formed on the enamel surface of the NaF + CPP-ACP group and the NaF group, making it smooth and uniform. On the surface of the TiF_4_ group, spherical deposits with larger diameters were found (Fig. 4A). As for dentin, the surface of the NaF + CPP-ACP group and the NaF group was more uniform than that of the DDW group, and the sediment was denser. The TiF_4_ group also had large-diameter spherical sediments (Fig. 4B).

The average surface roughness of each group is shown in Table 1. In enamel samples, there was no significant difference between all remineralized groups, but all remineralized groups had smoother surfaces than the demineralized group (*P* < 0.001). In dentin samples, the roughness was the lowest in the TiF_4_ group (98.87 ± 21.75 nm), followed by the NaF + CPP-ACP group (99.41 ± 36.49 nm). There was a significant difference between the two groups and the demineralization group (*P* < 0.05), but there was no significant difference between other treatment groups and the demineralization group (*P* > 0.05).

**Table 1:** Quantitative analysis of surface roughness of enamel and dentin in each group

	Enamel		Dentin	
	Roughness (Ra/µm)	*P* value	Roughness (Ra/µm)	*P* value
Demineralization	114.12 ± 36.30^a^	<0.001	145.63 ± 64.30^a^	< 0.05
DDW	63.49 ± 7.53^b^		123.83 ± 29.05^ab^	
NaF	54.75 ± 10.00^b^		128.51 ± 40.51^ab^	
CPP-ACP	63.47 ± 9.99^b^		135.85 ± 26.25^a^	
NaF + CPP-ACP	56.63 ± 4.28^b^		99.41 ± 36.49^b^	
TiF_4_	54.80 ± 12.46^b^		98.87 ± 21.75^b^	

All values are presented as means ± SD. The different letters indicate statistically significant differences

#### The morphology of dental hard tissues after remineralization

The results of SEM showed that compared with the DDW group, the enamel surface of all treatment groups had denser and more prominent deposits. The enamel surface deposits of the NaF + CPP-ACP group were the most dense and homogeneous, followed by the NaF group, and the CPP-ACP group was between the DDW group and the NaF group. The TiF_4_ group formed many large deposits, which were different from the other four groups (Fig. 4C).

For dentin samples, compared with the demineralized group, the dentinal tubules in the DDW group were partially opened, and similar conditions appeared in the dentinal tubules in the CPP-ACP group. The opening degree of dentinal tubules in TiF_4_, NaF + CPP-ACP, and NaF groups was limited, and more deposits appeared in most dentinal tubules. At the same time, except for the TiF_4_ group, the surface of dentin around the tube was smooth and uniform. Rough and uneven deposits were formed on the surface of dentin around the tube in the TiF_4_ group (Fig. 4D).

#### The changes of contents of Ca, P, F, and Ti elements at different depths after remineralization

**Fig. 5:**
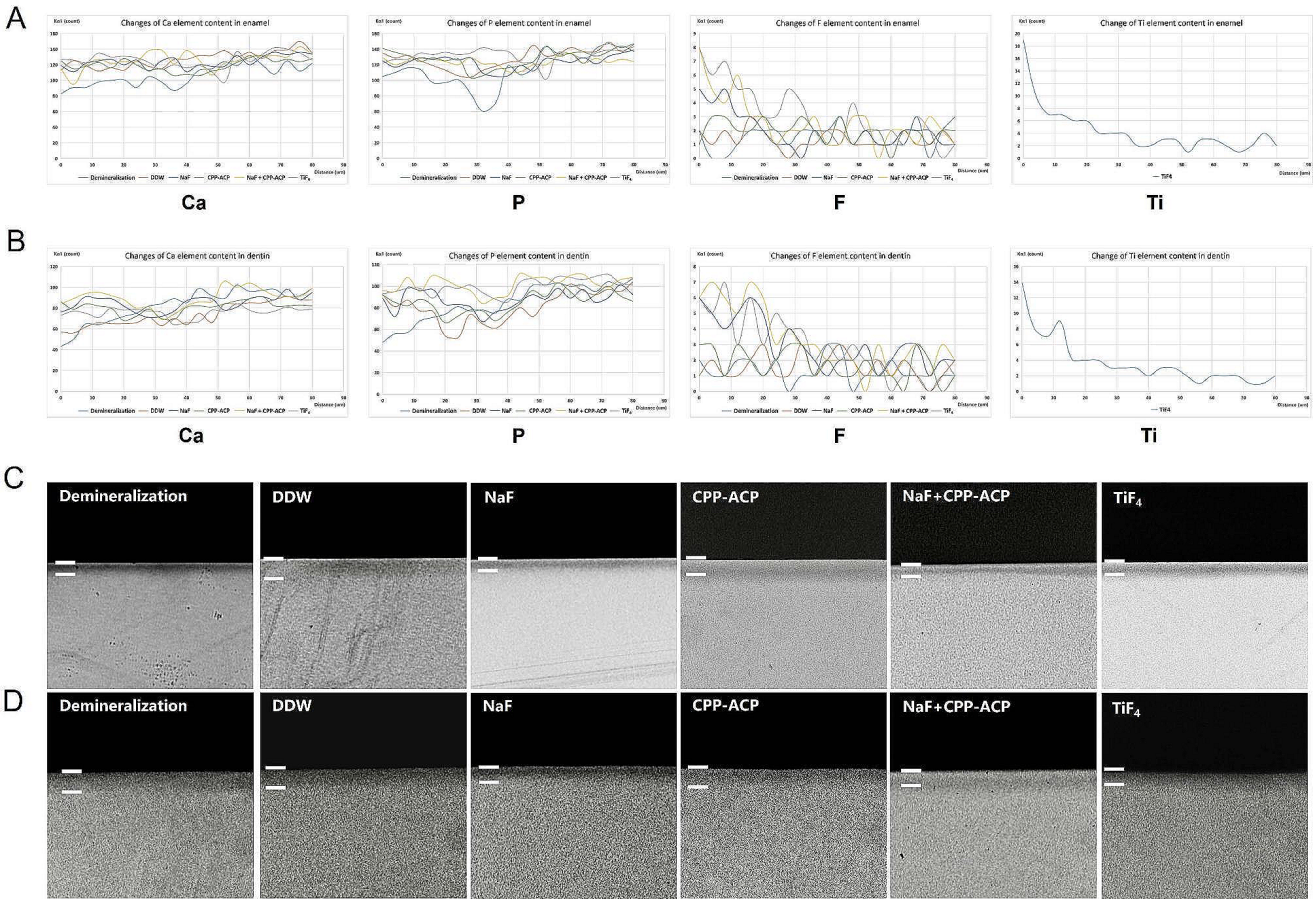
(**A**) The changes of Ca, P, F, and Ti elements content in irradiated enamel and (**B**) dentin at different depths after being treated with different remineralizing agents. (**C**) TMR images of demineralized enamel and (**D**) dentin treated with different remineralizing agents

EDS analysis of enamel samples (Fig. 5A) and dentin samples (Fig. 5B) showed that TiF_4_ had significantly accelerated Ca and P elements deposition. F element had penetrated up to 20 μm into the enamel lesion in the NaF + CPP-ACP group, while in the NaF group F element penetrated only 5 μm. However, no significant change in the F element was observed in the dentin lesion. In the TiF_4_ group, the Ti element had penetrated up to 20 μm into the lesion and 15 μm into the dentin lesion.

#### Analysis of changes in lesion depth

Representative TMR photos showed that, after TiF_4_, NaF, and NaF + CPP-ACP treatment, a remineralization layer with increased density can be observed on the enamel surface (Fig. 5C), but the remineralization layer in each group of dentin group is not obvious (Fig. 5D).

The results of the quantitative analysis of mineral loss and lesion depth in each group are shown in Table 2. In terms of lesion depth, there was no significant difference between all groups in enamel (*P* = 0.14) and dentin (*P* = 0.593). In enamel samples, mineral loss of the TiF_4_ group was 520 ± 225 vol% × µm, which was not significantly different from that of NaF + CPP-ACP and NaF groups (*P* > 0.05), but significantly lower than that of the other three groups (*P* < 0.05). In dentin samples, the mineral loss in the TiF_4_ group was the least (580 ± 225 vol% × µm), which had no significant difference with the NaF + CPP-ACP group (645 ± 195 vol% × µm) and the NaF group (740 ± 300 vol% × µm) but was significantly lower than that in the demineralization group, the DDW group, and the CPP-ACP group.

**Table 2:** Quantitative analysis of mineral loss and lesion depth of demineralized enamel and dentin treated with different remineralizing agents

	Enamel		Dentin	
	Mineral loss (Vol% × µm)	Lesion depth (µm)	Mineral loss (Vol% × µm)	Lesion depth (µm)
Demineralization	1885 ± 1023^a^	43.65 ± 28.6	900 ± 100^a^	57.90 ± 5.4
DDW	1025 ± 338^a^	40.45 ± 26.6	905 ± 340^a^	53.45 ± 17.9
NaF	780 ± 140^b^	45.75 ± 16.5	740 ± 300^b^	53.70 ± 6.7
CPP-ACP	1100 ± 478^a^	46.25 ± 15.5	900 ± 165^a^	61.75 ± 26.7
NaF + CPP-ACP	575 ± 215^b^	37.95 ± 10	645 ± 195^b^	54.65 ± 12.0
TiF_4_	520 ± 225^b^	44.95 ± 19.4	580 ± 225^b^	52.10 ± 11.0

All values are presented as means ± SD. The different letters indicate statistically significant differences

## Discussion

Based on the present results, our null hypotheses were rejected.

The radiation dose ranges from 60 Gy to 70 Gy in the treatment of HNC, which will be partially attenuated after reaching the dental hard tissues [39]. We explored the changes in microstructure and chemical composition of enamel and dentin after different doses of irradiation. In enamel, the XRD findings demonstrated that when the radiation dose reached 50 Gy, there was a gradual replacement of hydroxyapatite, the primary crystal constituent of enamel, by carbonate and other crystals. This observation was consistent with previous research conducted by Hedge et al. [40] and Qing et al. [41], where they reported similar trends. The decrease in crystallinity may have significant implications for the solubility of enamel and dentin, with potential effects on caries susceptibility [42]. As for dentin, when the radiation dosage reached 30 Gy, the dentin tubules began to partially occlude, and the dentin collagen fibers became sparse. When the radiation dose surpassed 50 Gy, nearly all dentin tubules were occluded, and the collagen fibers in the dentin became disordered and fractured. This result was similar to the finding of Walker et al. [43], whose result showed that high-dose radiation would lead to the disappearance of the dentin tubular structure. Taken together, it was found that both enamel and dentin were damaged when the radiation dose exceeded 50 Gy. Therefore, 50 Gy of radiation was selected for subsequent experiments.

Dentin displayed more noticeable changes than enamel in response to different radiation doses in our experiment. This is because there are higher organic content and water composition in dentin compared to enamel and the collagen matrix is highly reactive to radiation [44]. Radiation would change the secondary and tertiary structures of proteins and affect the hydration of collagen fibers, which could lead to the desiccation and fragility of dentin [16]. This explains why RRC is prone to occur in the cervical areas of teeth. Therefore, it is important to maintain the radiation dose below 30 Gy to decrease the risk of RRC in radiotherapy.

Radiation did not induce alterations in the elemental composition and distribution of dental hard tissues in the findings from EDS. To date, there is no available literature addressing radiotherapy causes effects at the atomic level, possibly because the radiation energy employed in radiotherapy falls significantly below the threshold required to induce atomic-level changes or migrations. Instead, it appears to primarily impact molecular composition and microstructural characteristics.

NaF and CPP-ACP both had been confirmed to have a remineralizing effect in the clinic [21, 25]. NaF not only binds with hydroxyapatite in dental hard tissues to form a more acid-resistant fluorohydroxyapatite [45], but also inhibits the adhesion, proliferation, and metabolism of cariogenic bacteria [46]. The remineralizing mechanism of CPP-ACP involves confining calcium ions, phosphate ions, and fluoride ions to the tooth surface simultaneously and continuously supplying calcium and phosphorus ions to the teeth [47]. In our study, samples were kept in a remineralization solution with rich calcium and phosphorus ions, rendering CPP-ACP ineffective and resulting in no significant difference. However, CPP-ACP can interact with fluoride ions, forming a novel cluster composed of amorphous calcium-fluoride-phosphorus. This composite not only maintains a high concentration of calcium ions, phosphate ions, and fluoride ions on the tooth surface but also prevents the rapid deposition of fluorapatite. It gradually releases the ions required for remineralization to increase the depth of remineralization [48]. This elucidates why the combined use of NaF and CPP-ACP in our study yielded superior remineralization effects compared to NaF alone or CPP-ACP alone group. This is consistent with the study by Llena et al., who indicated that the remineralization efficacy of Casein phosphopeptide-amorphous calcium fluoride phosphate (CPP-ACFP) surpasses that of fluoride varnish [49].

The morphology of dental hard tissues after remineralization was revealed by SEM and AFM images, in which the TiF_4_ group and NaF + CPP-ACP group showed better remineralization effects than others. In this research, the enamel surface became rough after radiation exposure and demineralization. Subsequently, after treatment with remineralizing agents and remineralization solution, depositions were observed on the enamel surface. Notably, the enamel surfaces in the NaF group and the NaF + CPP-ACP group displayed dense sedimentation, whereas the TiF_4_ group exhibited spherical deposits with larger diameters. Similar trends were observed in dentin samples. According to the findings of Büyükyilmaz et al. [27], the remineralization mechanism of TiF_4_ is based on the formation of an acid-resistant layer rich in titanium oxide and hydrated titanium phosphate on the tooth surface. This differs in terms of size and texture from the fluoro-hydroxyapatite induced by NaF or the mineral ions deposition during conventional remineralization [45]. The results of EDS in our experiment corroborated the accumulation of titanium on the surface of dental hard tissues after TiF_4_ treatment. Additionally, the results of AFM indicated that the remineralized sediments produced by the TiF_4_ group and NaF + CPP-ACP group were the most abundant and exhibited the lowest surface roughness following remineralization.

In EDS analysis, TiF_4_ greatly increased the depth of Ca, P, F, and Ti entering the demineralized enamel and dentin after radiation. In previous research results, compared with NaF varnish, TiF_4_ varnish was more effective in inhibiting enamel demineralization and promoting enamel remineralization [26, 46, 50]. This may be related to the low pH value of the TiF_4_ solution, which will lead to the dissolution of hydroxyapatite on the enamel surface and the dentin surface, forming a loose and porous structure, enabling calcium, phosphorus, and fluoride ions to enter deeper and obtain better remineralization effect [51]. Although the quantitative analysis of TMR in this study did not reveal a significant difference among the TiF_4_ group, the NaF group, and the NaF + CPP-ACP group, when considered in conjunction with TMR and EDS results, it became evident that the penetration depth of F element into enamel and dentin was significantly increased after treatment with TiF_4_ and NaF + CPP-ACP compared with NaF.

Based on our experimental results, TiF_4_ and NaF + CPP-ACP demonstrated a superior effect in preventing demineralization and promoting remineralization of enamel and dentin compared to other approaches. The protective effect of TiF_4_ was associated with the formation of an acid-resistant surface coating and increased fluoride absorption [52], it was perhaps more effective than NaF + CPP-ACP in preventing demineralization. However, the low pH value of TiF_4_ also means strict clinical use conditions. When high-concentration TiF_4_ is used for remineralization treatment, it is necessary to isolate the other teeth and soft tissues. In addition, we only used bovine teeth for remineralization experiments in vitro, more studies are needed to explore the clinical feasibility of these remineralizing agents in the future.

## Conclusions

In this study, the radiation dose within 70 Gy had minimal impact on the microstructure of enamel. However, radiation dose over 50 Gy would lead to the change of crystal phase in enamel. As for dentine, the radiation dose of 30 Gy could affect its microstructure, and the degree of dentin destruction was dose-dependent. In our remineralization experiment, CPP-ACP alone had a poor remineralization effect, but it could promote the remineralization of NaF synergistically. TiF_4_ and NaF + CPP-ACP both had a better remineralization effect on demineralized dental hard tissues after radiation than NaF alone. These findings will serve as a theoretical foundation for the prevention and treatment of RRC.

## Data Availability

The datasets used and/or analysed during the current study are available from the corresponding author on reasonable request.
